# Effects of Laparoscopic and Open Ovariectomy on Cortisol and Oxidative Stress in Dogs Under the Same Anesthesia Protocol

**DOI:** 10.3390/vetsci13040310

**Published:** 2026-03-24

**Authors:** Sandra Čechovičienė, Ieva Šidlauskaitė, Aidas Grigonis, Birutė Karvelienė, Ieva Sarapinienė, Ieva Čiapienė, Sigita Kerzienė, Vita Riškevičienė, Dalia Juodžentė

**Affiliations:** 1 Dr. L. Kriaučeliūnas Small Animal Clinic, Faculty of Veterinary, Veterinary Academy, Lithuanian University of Health Sciences, Tilžės Str. 18, 44307 Kaunas, Lithuaniadalia.juodzente@lsmu.lt (D.J.); 2 Institute of Cardiology, Lithuanian University of Health Sciences, Sukilėlių Ave 15, 50162 Kaunas, Lithuania; 3 Department of Animal Breeding, Faculty of Animal Sciences, Veterinary Academy, Lithuanian University of Health Sciences, Tilžės Str. 18, 44307 Kaunas, Lithuania; 4 Department of Veterinary Pathobiology, Faculty of Veterinary, Veterinary Academy, Lithuanian University of Health Sciences, Tilžės Str. 18, 44307 Kaunas, Lithuania

**Keywords:** cortisol, dog, laparoscopic, laparotomy, oophorectomy, pneumoperitoneum, surgical stress response

## Abstract

The main distinction between open and laparoscopic ovariectomy (OVE) is pneumoperitoneum (PNP) and anesthesia management. The objective of this study was to evaluate the effect of laparoscopic and open OVE on plasma cortisol, oxidative stress (OS), and cardiorespiratory parameters in dogs under the same anesthesia protocol. Twenty healthy female dogs were assigned to laparotomy OVE (LPTOVE, *n* = 10) and laparoscopic OVE (LAPOVE, *n* = 10) groups. OS was assessed using the total oxidant status (TOS), total antioxidant status (TAS), and oxidative stress index (OSI). Blood samples were collected prior to sedation (T0); prior to surgical incision (T1); prior to surgical closure in the LPTOVE group; and prior to the deflation of CO_2_ in the LAPOVE group (T2), 2 h after the surgery (T3). The monitored cardiorespiratory parameters were heart rate, respiratory rate, and end-tidal CO_2_ (EtCO_2_). Cortisol levels at T3 were markedly elevated in the LPTOVE group compared to the LAPOVE group. No substantial changes in TOS, TAS, or OSI between groups were detected. In the LAPOVE group, TOS diminished at T2, but TAS declined and OSI escalated at T3. EtCO_2_ levels were elevated in the LAPOVE group at the end of the procedure. These findings support laparoscopic OVE as a less traumatic alternative to open surgery, with careful management of PNP and anesthesia recommended to minimize postoperative stress.

## Simple Summary

Laparoscopic and open ovariectomy have been widely compared. Although laparoscopic surgery is associated with a lower rate of intraoperative and postoperative complications, its systemic effects, particularly those related to pneumoperitoneum, remain insufficiently understood. The aim of this study was to evaluate the effects of laparoscopic and open ovariectomy on plasma cortisol levels, oxidative stress, and cardiorespiratory parameters. The results of the current study suggest that laparoscopic ovariectomy is less traumatic and painful than open ovariectomy. However, the postoperative alterations in oxidative stress indicate that certain components of the stress response following laparoscopic and open procedures may be similar. In addition, the increased end-tidal CO_2_ observed at the end of laparoscopic ovariectomy suggests altered respiratory dynamics, potentially related to pneumoperitoneum and anesthesia management. The observed increases in cortisol levels and oxidative stress during open ovariectomy suggest a pronounced stress response, potentially related to anesthetic management. In conclusion, laparoscopic ovariectomy may be preferred due to its lower stress response. However, a thorough understanding of the systemic effects of pneumoperitoneum is essential for adequate perioperative management.

## 1. Introduction

Ovariectomy (OVE) represents one of the most frequently performed surgical interventions in canine patients [1,2]. The main reasons for neutering dogs include population control, preventive health care, and behavioral management [3]. In recent decades, the adoption of minimally invasive surgical techniques, particularly laparoscopy, has significantly risen in Veterinary Medicine, demonstrating enhanced success rates, and introducing various methodologies for procedures like ovariohysterectomy (OHE) and ovariectomy (OVE) [4,5,6,7,8,9].

Multiple previous studies evaluating the main advantages of laparoscopic neutering procedures compared with conventional open techniques have focused on intraoperative and postoperative complications. Most authors have reported reduced tissue trauma associated with smaller incisions, as well as a lower incidence of postoperative complications in laparoscopic surgery [10,11,12,13]. Furthermore, laparoscopic approaches have been associated with lower rates of intraoperative bleeding, reduced perioperative pain, decreased infection rates and morbidity, and shorter durations of hospitalization [10,13,14,15,16]. Although laparoscopic surgery is associated with lower intraoperative and postoperative complication rates, its systemic effects are not yet fully understood.

The main difference between open and laparoscopic surgery is the establishment of pneumoperitoneum (PNP), achieved by CO_2_ insufflation, which is necessary for the creation of an adequate surgical field. PNP is associated with elevated intra-abdominal pressure (IAP), which causes cranial displacement of the diaphragm and induces changes in ventilatory and cardiovascular function [17,18,19,20]. Moreover, one study suggested that CO_2_ insufflation and its absorption through the abdominal cavity may contribute to acid–base imbalance and cardiorespiratory disturbances [21]. Furthermore, higher IAP has been associated not only with these disturbances but also with increased oxidative stress (OS) [19,20,22]. Lee and Choi reported that, in dogs, the total antioxidant status (TAS) decreased and the oxidative stress index (OSI) increased at 90 min and 24 h after CO_2_ PNP deflation when IAP was raised to 15 mmHg for 60 min, whereas no significant changes occurred at 12 mmHg [22].

Reactive oxygen species (ROS) are integral to normal cellular metabolism, contributing to pathogen defense and playing a key role in inflammatory processes. However, excessive ROS production and an imbalance between pro-oxidant and antioxidant mechanisms within the organism can lead to OS [23,24,25,26]. Excessive oxidants delay wound healing by exerting cytotoxic effects on dermal fibroblasts and keratinocytes, inactivating antioxidant defenses, and promoting the chemotaxis of inflammatory cells [26,27,28]. OS has been used as a marker for assessing the impact of surgical techniques on the organism [18,29,30,31,32,33]. Most previous studies indicate that laparoscopic OVE induces less OS than open OVE. However, one study reported no difference between the techniques. Lee et al. reported that both surgical approaches induced oxidative stress, characterized by an increased total oxidant status (TOS) and OSI, and decreased TAS after incision closure. However, TOS levels were significantly lower in the laparoscopic group than in the open surgery group [18]. Costa et al. observed significantly higher OS marker levels at 24 h and 48 h postoperatively in the open surgery group, characterized by increased malondialdehyde (MDA) and myeloperoxidase (MPO) and decreased butyrylcholinesterase (BuChE), whereas only mild changes were observed in the laparoscopic group [30]. The longest follow-up of OS, at 168 h (7 days) postoperatively, was reported by Naghavi et al.; the malondialdehyde (MDA) and total antioxidant capacity (TAC) levels were significantly higher in the celiotomy group compared with the laparoscopic OVE [33]. In addition, the severity of surgical intervention has been shown to correlate positively with OS [34].

Plasma cortisol levels are commonly utilized as a biochemical indicator to evaluate surgical stress and pain in both Human and Veterinary Medicine [10,14,35,36]. Numerous studies evaluating alterations in plasma cortisol levels subsequent to laparoscopic or open ovariohysterectomy/ovariectomy have predominantly concentrated on the postoperative phase, during which cortisol concentrations have often been elevated following open surgery [10,14,37,38,39]. Nevertheless, the intraoperative evaluation of plasma cortisol concentrations is infrequently documented [35,39]. Alipour et al. used four distinct protocols to test cortisol levels before and 40 min after CO_2_ insufflation into the abdomen while preserving anesthesia. No significant differences between the groups were found. However, cortisol levels were significantly higher 40 min after PNP compared with baseline values within each group [35].

Most previous studies have focused on the chronic effects of cortisol on OS in dogs with endocrine disorders such as spontaneous hypercortisolism and Cushing’s disease [40,41,42,43]. The relationship between cortisol levels and oxidative stress in dogs undergoing surgical procedures, a model of acute stress, remains poorly understood. In other species, the experimental manipulation of cortisol has been shown to influence antioxidant systems. Kim et al. reported that cortisol administration in juvenile brown trout increased the levels of the natural antioxidant glutathione, suggesting a potential role in maintaining redox balance under acute stress conditions [44]. Although direct extrapolation to dogs is limited due to species differences, these findings indicate that cortisol may similarly modulate antioxidant defenses during acute stress in clinical settings.

A review of the scientific literature indicates that plasma cortisol and oxidative stress in the comparison of laparoscopic and open OVE have mostly been measured postoperatively. Therefore, there is a research gap regarding the evaluation of these parameters and their intraoperative effects. To assess the oxidative stress status in dogs undergoing laparoscopic and open OVE, the plasma total oxidant status (TOS), total antioxidant status (TAS), and oxidative stress index (OSI) were measured. The TOS is a marker reflecting the cumulative level of oxidants in the plasma, whereas the TAS represents the plasma’s antioxidant capacity and its ability to scavenge free radicals [24,45]. The OSI represents the balance between oxidants and antioxidants, calculated as the ratio between the TOS and TAS [18,22,24,34,45,46,47].

The aim of this study was to evaluate the effects of laparoscopic and open ovariectomy on plasma cortisol levels, oxidative stress, and cardiorespiratory parameters in healthy dogs under the same anesthesia protocol.

## 2. Materials and Methods

### 2.1. Animals

This clinical study was conducted in the Lithuanian University of Health Sciences Veterinary Academy Dr. L. Kriauceliunas Small Animal Clinic. The animal study protocol was approved by the Ethics Committee of the Lithuanian University of Health Sciences (protocol code Nr. 2024-BEC3-T-009, approved on 12 March 2024). All the owners signed an informed consent form before enrolling their dogs in the study.

A total of 20 client-owned healthy female dogs, scheduled for routine neutering procedures, were included in this study. For inclusion, all dogs had to be in the anestrus phase of the estrous cycle and classified as ASA (American Society of Anesthesiologists) I, based on an anamnesis, physical examination, complete blood cell count, and analysis of serum biochemistry. The animals were assigned to two groups according to the owners’ decisions regarding the surgical technique: the laparotomy ovariectomy group (LPTOVE, *n* = 10) and laparoscopic ovariectomy group (LAPOVE, *n* = 10). The dogs in the LPTOVE group were 28.90 ± 7.54 months old and weighed 11.53 ± 2.18 kg. All the dogs in this group were mixed-breed. The dogs in the LAPOVE group were 23.00 ± 4.72 months old and weighed 10.24 ± 1.31 kg. This group consisted of seven mixed-breed dogs, one Dachshund, one Standard Poodle, and one Border Collie.

### 2.2. Anesthesia and Analgesia

All dogs were put under general anesthesia using the same anesthesia protocol after a 12 h fast. For sedation, both groups received 0.5 mg kg^−1^ methadone (Richter Pharma AG, Wels, Austria) and 3 µg kg^−1^ dexmedetomidine (Orion Corporation, Espoo, Finland) intramuscularly. After the injection of sedatives, the dogs were then kept in the same quiet room for 15 min under the direct supervision of an anesthesiologist. An intravenous catheter was placed in the cephalic vein 15 min after sedation, and anesthesia was then induced with propofol (Accord Healthcare B.V., Utrecht, The Netherlands) at 1 to 2 mg kg^−1^ until the palpebral and gag reflexes were absent. When no palpebral and gag reflexes were observed, a tracheal tube, based on the animal’s size, was placed. Anesthesia was maintained with sevoflurane (SVO; AbbVie S.r.l., Campoverde di Aprilia (LT), Italy) (vaporizer 2.00%) in pure oxygen (1.00 L min^−1^). A 0.9% sodium chloride solution (Braun Melsungen AG, Melsungen, Germany) was infused at a flow rate of 5 mL kg^−1^ per hour with an infusion pump (Braun, Melsungen, Germany), starting 15 min after sedation and continuing up to full recovery from anesthesia. In the LAPOVE group, animals were ventilated using a volume-controlled ventilation mode following the induction of PNP, and maintained until CO_2_ deflation. The tidal volume (Vt) was calculated as follows: Vt (ml) = 10 × animal weight (kg). The inspiratory-to-expiratory (I:E) ratio was set at 1:3 for dogs weighing <10 kg and at 1:2 for dogs weighing ≥10 kg. The airway pressure limit was set to 20 cmH_2_O. The anesthesia time was defined as the time from sedation until the discontinuation of the inhalation of SVO. After the end of SVO inhalation, 30 µg kg^−1^ atipamezole (Eurovet Animal Health B.V., Bladel, The Netherlands) was administered subcutaneously as a dexmedetomidine antagonist. Intramuscular buprenorphine at 0.015 mg kg^−1^ (Richter Pharma AG, Wels, Austria) and subcutaneous meloxicam at 0.2 mg kg^−1^ (Chanelle Pharmaceuticals Manufacturing Ltd., Loughrea, Co., Galway, Ireland) were administered to all dogs after stopping SVO inhalation.

The anesthesia depth and animals’ vital parameters were continuously monitored every 5 min by an anesthesiologist. The heart rate (HR; beats min^−1^); respiratory rate (RR; breaths min^−1^); systolic arterial pressure (SAP; mmHg); diastolic arterial pressure (DAP; mmHg); oxygen saturation (SpO_2_; %); and end-tidal carbon dioxide (EtCO_2_; mmHg) were monitored by using a veterinary patient monitoring system (iM8 VET, Edan, Germany). Body temperature (BT; °C) was measured using an electric thermometer. SAP and DAP were measured using a noninvasive oscillometric method. SpO_2_ was measured using a pulse oximeter (OXY-100 VET, Gima, Fara d’Adda, Italy), and EtCO_2_ was measured using a capnograph, installed into an anesthesia machine (WATO EX-35, Mindray, Shenzhen, China). An electronic circulating warm heating pad (Shenzhen Anpan Health Industry Co., Ltd., Shenzhen, Guangdong, China) was used to maintain BT between 38.00 and 39.30 °C. The vital parameters were first evaluated 15 min after methadone and dexmedetomidine administration and continuously monitored until the end of anesthesia.

### 2.3. Surgical Procedure

Animals were placed in dorsal recumbency. Trichotomy at the surgical site and aseptic abdominal skin preparation were performed in both groups. Laparotomy OVE and laparoscopic OVE were performed by the same veterinary surgical team. The surgery time was defined as the time from skin incision to skin closure.

For the LPTOVE group, laparotomy ovariectomy was performed. A midline celiotomy was performed caudal to the umbilicus. The length of the incision was estimated by the surgeon depending on the size of the animal and extended during surgery if it was necessary according to the following criteria: the suspensory ligaments and both ovarian pedicles had to be ligated with as little traction as possible. A skin incision was made with a No. 11 scalpel blade (AMBRO, Naples, Italy), and subcutaneous tissues were dissected at the midline and separated from the abdominal fascia with Mayo scissors (KLS Martin SE & Co. KG., Tuttlingen, Germany). Bleeding was controlled using bipolar electrosurgical equipment (KLS Martin SE & Co. KG., Tuttlingen, Germany). The left ovary was identified after left uterine horn identification using a spay hook (KLS Martin SE & Co. KG., Tuttlingen, Germany). Subsequently, the left ovary was clamped with hemostatic forceps (KLS Martin SE & Co. KG., Tuttlingen, Germany) at the suspensory ligament and dissected from the surrounding tissues using a bipolar electrosurgical vessel-sealer device (KLS Martin SE & Co. KG., Tuttlingen, Germany). The same procedure was performed with the right ovary. After assessing the absence of bleeding, the abdominal incision was closed. The abdominal fascia and subcutaneous layer were closed using a simple continuous pattern, and the skin was closed with an intradermal pattern, both with synthetic absorbable monofilament polydioxanone suture material (Surgicryl^®^, SMI AG, Saint-Vith, Belgium).

The LAPOVE surgeries were performed using a 2-portal technique. The video monitor was placed at the cranial end of the dog. In each dog, the first 10 mm trocar and cannula assembly (VersaOne™, COVIDIEN™, North Haven, CT, USA) was placed 1 cm caudal to the umbilicus in a midline of the abdomen using the modified Hasson technique [48]. Pneumoperitoneum (PNP) was established with CO_2_ insufflation (DF-32O Automatic Insufflator, Micon Medizintechnik GmbH, Kaltenkirchen, Germany), maintaining an intra-abdominal pressure of 10–12 mmHg throughout the procedure. A 5 mm, 30°, 30 cm long laparoscope (Dr. Fritz, Dr. Fritz Endoscopes GmbH, Buchheim, Germany) was inserted through the first cannula, and the abdominal cavity was inspected. The second trocar (10 mm) was placed paramedially to the first trocar, and the optic was transferred from the caudal to the cranial cannula [4]. Each ovary was clamped with grasping forceps at the opening of the ovarian bursa through the caudal cannula and secured to the abdominal wall and suspensory ligament using 1-0 polydioxanone suture. Subsequently, the ovaries were dissected from the surrounding tissues through the caudal cannula using a bipolar electrosurgical vessel-sealing device (KLS Martin SE & Co. KG., Tuttlingen, Germany) and removed from the abdominal cavity through the same cannula with grasping forceps. After the deflation of CO_2_ and removal of the cannulas, the abdominal incisions were closed using the same suturing technique as in the LPTOVE group.

### 2.4. Analysis of Blood Samples

**Figure 1:**
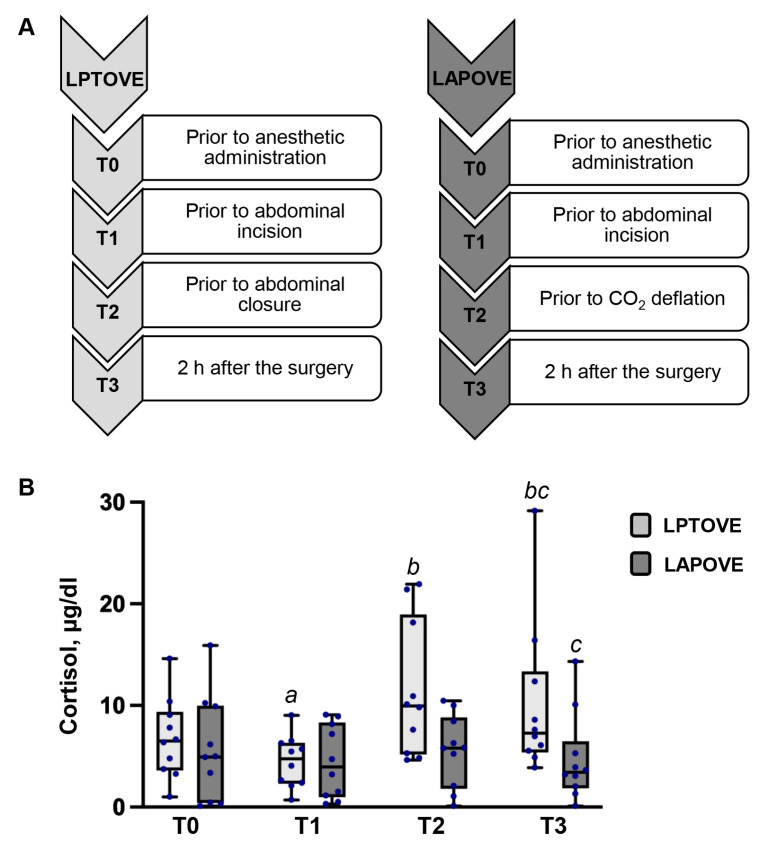
(**A**) Timeline of blood sample collection. (**B**) Cortisol alterations in the dogs undergoing laparoscopic and open ovariectomy at different time points of the study. Values are expressed as medians and interquartile ranges [IQR: 25th–75th percentiles]. Definitions of time points (T0–T3) are provided in Scheme (**A**). *a* indicates significant differences from T0 within the LPTOVE group (*p* < 0.05). *b* indicates significant differences from T1 within the LPTOVE group (*p* < 0.05). *c* indicates significant differences between the LPTOVE and LAPOVE groups at the same time point T3 (*p* < 0.05).

Venous blood samples (2.00 mL per sample) were collected into heparin tubes via venipuncture from the jugular vein. Blood samples were taken at four time points: T0—serving as the reference baseline, prior to sedation with the administration of drugs; T1—prior to surgical incision of the abdominal wall; T2—just before closing the abdominal incision in the LPTOVE group and prior to the deflation of CO_2_ in the LAPOVE group; T3—at 2 h after the surgery (Figure 1). Plasma was separated by centrifugation at 1500 rpm for 15 min within 30 min of sample collection. An automated biochemical analyzer (FUJIFILM DRI-CHEM IMMUNO AU10V, FUJIFILM Corporation, Tokyo, Japan) was used to evaluate cortisol levels, using 100 µL of plasma per sample. The cortisol reference values ranged from 0.2 to 5.9 µg dl^−1^. The remaining plasma was stored at −80 °C until analysis for the total oxidant status (TOS) and total antioxidant status (TAS), but no longer than 6 months. Plasma samples were thawed only once prior to analysis of the TOS and TAS, and no hemolysis was observed in any sample.

The TOS and TAS were determined using a Tecan Infinite^®^ 200 PRO plate reader (Tecan Trading AG, Männedorf, Switzerland) and Colorimetric Assay kits (Thermo Fisher Scientific, Waltham, MA, USA) following the manufacturers’ instructions. Each sample was measured in duplicate. The TOS assay had a sensitivity of 2.5 μmol H_2_O_2_ Equiv./L, with intra-assay and inter-assay coefficients of variation of 2.3% and 3.5%, respectively. The TAS assay had a sensitivity of 0.23 mmol Trolox Equiv./L, with intra-assay and inter-assay coefficients of variation of 4.6% and 7%, respectively.

The TOS was measured using a colorimetric method based on the oxidation of a chromogenic substrate. A 20 μL volume of plasma sample was added to each sample well. Then, 200 μL of chromogenic agent was added and thoroughly mixed using a microplate reader for 5 s, and the optical density (OD) of each well was measured at 590 nm and recorded as A_1_. Subsequently, 50 μL of substrate solution was added to each well, mixed for 5 s, and incubated at 37 °C for 5 min. The OD was then measured again at 590 nm and recorded as A_2_. The change in absorbance (∆A = A_2_ − A_1_) was used for TOS calculation as follows: TOS (μmol H_2_O_2_ Equiv./L) = (∆A_590_ − b) ÷ a × f, where ∆A_590_ = ∆A_sample − ∆A_blank (∆A_blank is the change in the absorbance of the blank when the standard concentration is 0), a is the slope and b is the intercept of the standard curve, and f is the dilution factor of the sample prior to analysis. For the standard curve, a 200 μmol/L H_2_O_2_ standard solution was serially diluted to 0, 10, 20, 40, 50, 60, 80, and 100 μmol/L, and 20 μL of each dilution was added in duplicate to the wells. The duplicate readings were averaged, and the mean ∆A of the blank was subtracted to obtain absolute ∆A values. The resulting standard curve for TOS was linear (y = 0.005x + 0.0266, R^2^ = 0.9764), with the absolute ∆A plotted on the y-axis and H_2_O_2_ concentration on the x-axis.

The TAS was measured using a colorimetric method based on the oxidation of ABTS (2,2′-azino-bis(3-ethylbenzothiazoline-6-sulfonic acid)). A 10 μL volume of plasma sample was added to each well, followed by 200 μL of buffer. The OD was measured at 660 nm and recorded as A_1_. Then, 20 μL of chromogenic agent was added, mixed by pipetting 5–6 times, and incubated at 37 °C for 5 min. The OD was measured again at 660 nm (A_2_), and the change in absorbance (∆A = A_2_ − A_1_) was used for TAS calculation as follows: TAS (mmol Trolox Equiv./L) = (∆A_blank − ∆A_sample − b) ÷ a × f, where ∆A_blank is the change in the absorbance of the blank when the standard concentration is 0, a and b are the slope and intercept of the standard curve, and f is the dilution factor of the sample prior to analysis. For the standard curve, a 2 mmol/L Trolox standard solution was serially diluted with 60% ethanol to obtain concentrations of 0, 0.4, 0.8, 1.2, 1.4, 1.6, 1.8, and 2 mmol/L. A 10 μL volume of each dilution was added in duplicate to the wells, and the duplicate readings were averaged. The mean ∆A value of the blank was subtracted from all standard readings to obtain the absolute ∆A values. The resulting standard curve for TAS was linear (y = 0.4057x + 0.034, R^2^ = 0.9956), with the absolute ∆A plotted on the y-axis and Trolox concentration on the x-axis.

The oxidative stress index (OSI) was calculated as follows: OSI (arbitrary unit) = TOS (µmol H_2_O_2_ Eq L^−1^)/TAS × 10 (mmol Trolox Eq L^−1^) [46].

### 2.5. Statistical Analysis

Data normality was evaluated by the Shapiro–Wilk test. The majority of the data were not distributed normally; therefore, the data are described using the median and interquartile range [IQR: 25th–75th percentiles]. Statistical analysis was performed with the Mann–Whitney U, Wilcoxon Signed Rank, and Spearman‘s tests. The data were considered statistically significant if the *p* value < 0.05. Calculations were performed by using IBM SPSS Statistics 29.0.2.0 software (IBM Corp., Armonk, NY, USA).

## 3. Results

Both the LAPOVE and LPTOVE groups were matched for age (24 [IQR: 11.8–40.5] and 24 (IQR: 10.8–24.0] months, respectively) and weight (11.8 [IQR: 5.0–15.5] and 9.2 [IQR: 6.9–12.8] kg, respectively). No significant differences in anesthesia or surgery time between the groups were observed. The laparotomy ovariectomy lasted for 60 [IQR: 50–65] min, and the laparoscopic ovariectomy lasted for 68 [IQR: 60–85] min. The anesthesia time (T0–T3) in the LAPOVE and LPTOVE groups lasted for 100 [IQR: 100–125] and 100 [IQR: 80–100] min, respectively. The initial physiological and vital parameters before the procedure did not statistically differ in both animal groups. All dogs were stable throughout the procedure and completed the study.

### 3.1. Cortisol Evaluation

The effects of LPTOVE and LAPOVE on the plasma cortisol concentration at different time points (T0–T3) are demonstrated in Figure 1. In the LPTOVE group, the cortisol concentration decreased significantly from 6.51 [IQR: 3.73–9.05] µg dl^−1^ at T0 to 4.76 [IQR: 2.38–6.29] µg dl^−1^ at T1 (*p* = 0.007). The cortisol level before closing the abdominal incision (T2) increased significantly by 51% compared to the cortisol concentration before surgical incision (from 4.76 [IQR: 2.38–6.2] µg dl^−1^ to 9.97 [IQR: 5.30–18.16] µg dl^−1^) (*p* = 0.028). At T3, the cortisol concentration (7.29 [IQR: 5.54–12.36] µg dl^−1^) significantly increased in comparison to T1 (*p* = 0.013) and did not significantly change compared to the control value. In addition, the cortisol concentration increased at T0, T2, and T3 by 9%, 41%, and 19%, respectively, compared to the upper limit of the reference range. In the LAPOVE group, cortisol levels did not show any significant differences across the T0–T3 time points, and all values remained within the reference range. The comparative analysis revealed significant difference between the LPTOVE and LAPOVE groups at T3. The cortisol concentration in the LPTOVE group animals was 53% higher than that in the LAPOVE group (7.29 [IQR: 5.54–12.36] µg dl^−1^ and 3.43 [IQR: 2.04–5.28] µg dl^−1^, respectively) (*p* = 0.016).

### 3.2. Oxidative Stress Evaluation

**Figure 2:**
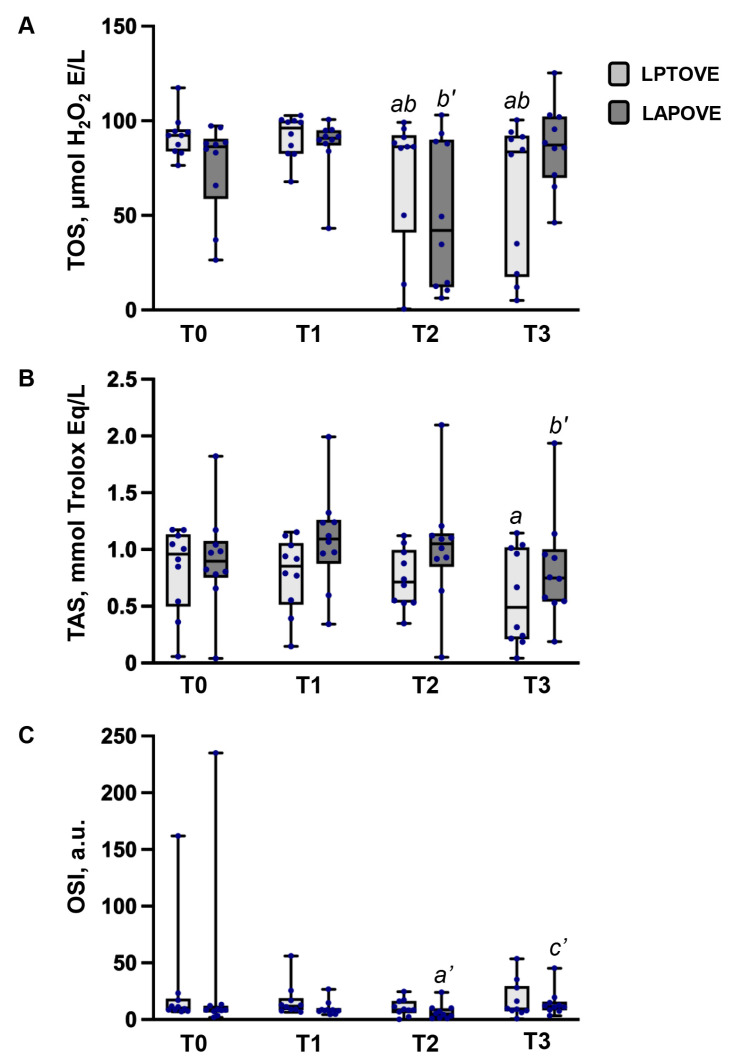
OS alterations in the dogs undergoing laparoscopic and open ovariectomy at different time points of the study: (**A**) total oxidant status (TOS), (**B**) total antioxidant status (TAS), and (**C**) oxidative stress index (OSI). Values are expressed as medians and interquartile ranges [IQR: 25th–75th percentiles]. (T0)—blood sample taken prior to sedation; (T1)—blood sample taken prior to surgical incision; (T2)—prior to surgical closure in the LPTOVE group, and prior to the deflation of CO_2_ in the LAPOVE group; (T3)—blood sample taken 2 h after the surgery. *a* indicates significant differences from T0 within the LPTOVE group (*p* < 0.05). *a’* indicates significant differences from T0 within the LAPOVE group (*p* < 0.05). *b* indicates significant differences from T1 within the LPTOVE group (*p* < 0.05). *b’* indicates significant differences from T1 within the LAPOVE group (*p* < 0.05). *c’* indicates significant differences from T2 within the LAPOVE group (*p* < 0.05).

Oxidative stress parameters (TOS, TAS, and OSI) were assessed at four time points (T0–T3) in both animal groups (Figure 2). In the LPTOVE group, the TOS decreased significantly at T2 (8.31 [IQR: 50.06–91.25] μmol H_2_O_2_ Eq L^−1^) and T3 (83.52 [IQR: 19.20–91.55] μmol H_2_O_2_ Eq L^−1^) by 6% and 9%, respectively, compared to T0 (92.37 [IQR: 83.98–94.44] μmol H_2_O_2_ Eq L^−1^) (*p* = 0.047, *p* = 0.028, respectively). Similarly, the TOS decreased significantly at T2 and T3 compared to T1 (96.17 [IQR: 82.66–100.0] μmol H_2_O_2_ Eq L^−1^) (*p* = 0.037, *p* = 0.037, respectively). The TAS decreased significantly at T3 (0.49 [IQR: 0.22–1.01] mmol Trolox Eq L^−1^) by 50% compared to T0 (0.96 [IQR: 0.54–1.12] mmol Trolox Eq L^−1^) (*p* = 0.037). No significant differences in OSI were observed between the T0–T3 time points. In the LAPOVE group, the TOS decreased significantly at T2 (42.06 [IQR: 12.54–89.04] μmol H_2_O_2_ Eq L^−1^) by 54% compared to T1 (90.83 [IQR: 87.96–94.88] μmol H_2_O_2_ Eq L^−1^) (*p* = 0.022). In contrast, the TAS decreased significantly at T3 (0.75 [IQR: 0.54–0.96] mmol Trolox Eq L^−1^) by 32% compared to T1 (1.10 [IQR: 0.97–1.24] mmol Trolox Eq L^−1^) (*p* = 0.007). The OSI decreased significantly by 43% at T2 compared to the control value (from 8.47 [IQR: 7.26–12.09] to 4.82 [IQR: 1.29–10.03] arbitrary units) (*p* = 0.013). However, the OSI increased significantly by 58% at T3 compared to T2 (from 4.82 [IQR: 1.29–10.03] to 11.47 [IQR: 8.48–14.85] arbitrary units) (*p* = 0.037). The comparative analysis revealed no significant difference between the LPTOVE and LAPOVE groups in TOS, TAS, and OSI levels at the T0–T3 time points.

### 3.3. Correlation Between Cortisol and Oxidative Stress Parameters

The correlation between cortisol levels and the TOS, TAS, and OSI was assessed (Table 1). In the LPTOVE group, the cortisol concentration at T1 and TOS at T2 were significantly and strongly negatively correlated (r = −0.673, *p* = 0.033). Furthermore, a significant and strong negative correlation between cortisol levels and OSI at T2 was observed (r = −0.721, *p* = 0.019). No correlation between cortisol and the TAS was observed at any time point in the LPTOVE group. In the LAPOVE group, no correlation between cortisol and oxidative stress parameters was observed at any time point.

### 3.4. Cardiorespiratory Parameter Evaluation

The HR, RR, and EtCO_2_ values are demonstrated in Table 2, Table 3 and Table 4. HR and RR were continuously monitored during the procedure starting 15 min after sedation until the end of anesthesia. In both the LPTOVE and LAPOVE groups, HR remained at baseline values, with no significant differences between the groups observed (Table 1). In the LPTOVE group, HR increased significantly at t = 45 min (70 [IQR: 60–72] bpm) and remained elevated until the end of the inhalation of SVO compared to t = 15 min (60 [IQR: 54–65]) (*p* < 0.05). Similarly, in the LAPOVE group, HR increased significantly at t = 35 min (70 [IQR: 64–80] bpm) and continued to increase until the end of anesthesia compared with t = 15 min (62 [IQR: 60–76] bpm) (*p* < 0.005).

In both the LPTOVE and LAPOVE groups, RR remained at baseline (Table 3). The comparative analysis revealed significant differences between the LPTOVE and LAPOVE groups at t = 15 min and t = 20 min: RR in the LPTOVE group was 23% and 17% higher, respectively, compared to the RR values in the LAPOVE group (*p* = 0.009, *p* = 0.008, respectively). Similarly, in the LPTOVE group, RR was 17% and 23% higher at t = 45 min and t = 50 min, respectively, compared with the corresponding time points in the LAPOVE group (*p* = 0.049, *p* = 0.036, respectively). In the LPTOVE group, no significant differences in RR were observed during the anesthesia duration, whereas in the LAPOVE group, RR increased significantly at t = 50 min (10 [IQR: 10–12] bpm) and remained elevated until the end of anesthesia compared to t = 15 min (10 [IQR: 10–10] bpm) (*p* < 0.005).

End-tidal carbon dioxide (EtCO_2_) was recorded starting 20 min after sedation and continued until the end of anesthesia (Table 4). The comparative analysis of EtCO_2_ between groups revealed significant differences at the final three time points of anesthesia. In the LAPOVE group, EtCO_2_ was higher at t = 1 h 00 min (50.5 [IQR: 49.0–52.0] mmHg); t = 1 h 05 min (51.5 [IQR:49–52] mmHg); and t = 1 h 10 min (51 [IQR: 50–52] mmHg) by 19%, 20%, and 18%, respectively, compared to the EtCO_2_ values in the LPTOVE group (42.5 [IQR: 39.0–48.0] mmHg; 43.0 [IQR: 39.0–47.0] mmHg; and 43.0 [IQR: 39.0–46.0] mmHg, respectively) (*p* = 0.002, *p* < 0.001, *p* < 0.001, respectively). No significant within-group differences were detected.

## 4. Discussion

Laparoscopic and open OVE have been extensively compared. Most studies have focused on surgical techniques, anesthesia management, postoperative complications, and pain assessment. However, there is limited understanding of the laparoscopic technique, particularly regarding the establishment of PNP and its effects on systemic changes during the intraoperative period.

Most studies assessing changes in plasma cortisol concentrations associated with laparoscopic and open OHE or OVE have evaluated cortisol levels during the postoperative period [10,14,37,38,39]. Previous studies have shown increased plasma cortisol levels at 2 h after surgery in the ovariohysterectomy groups compared with laparoscopic-assisted ovariohysterectomy groups [10,14]. Furthermore, Sherin et al. found that the plasma cortisol concentration was significantly higher 4 h postoperatively in the OVE group compared with the laparoscopic OVE group [39]. In the present study, consistent with previous reports, plasma cortisol levels were significantly higher at 2 h postoperatively (T3) in the LPTOVE group compared with the LAPOVE group. The plasma cortisol concentration was 53% higher at T3 in the LPTOVE group than in the LAPOVE group.

To the best of our knowledge, there are few published studies measuring plasma cortisol levels before CO_2_ deflation during laparoscopic OVE or before abdominal wall incision and closure in open OVE [35,39]. In our study, cortisol levels in the LPTOVE group were significantly higher at T2 (prior to abdominal closure) than at T1 (prior to abdominal wall incision), with an increase of 51%, whereas cortisol levels at the same time points did not significantly differ in the LAPOVE group. Furthermore, in the LPTOVE group, cortisol levels at T2 exceeded the upper limit of the reference range by 41%, while remaining within the normal range in the LAPOVE group. The plasma cortisol concentration has been used as a biochemical marker for assessing surgical stress and pain in dogs [10,14,35]. The results of this study suggest increased surgical stress towards the end of open ovariectomy, potentially related to the more invasive nature of the procedure. In contrast, laparoscopic OVE provoked a smaller response to surgical stress, possibly due to the less invasive nature of the laparoscopic approach.

Most previous studies indicate that laparoscopic OVE induces less OS than open OVE [18,30,31,32,33]. In this study, the TOS decreased at T1 and T2 in both groups. However, the decrease was mild in the LPTOVE group, whereas in the LAPOVE group, the TOS at T2 decreased by 51% compared with the TOS at T1. Similarly, the TAS decreased mildly at T1 and T2 in the LAPTOVE group, whereas a mild increase was observed in the LAPOVE group. Timing is an important consideration when interpreting these findings, as oxidative stress responses are highly dynamic. The earliest postoperative sampling point may have been insufficient to capture the peak oxidative burst, and measurements obtained immediately after surgery likely reflect an early physiological response rather than the subsequent inflammatory phase, during which ROS production is typically more pronounced [49,50]. Another factor may relate specifically to laparoscopic surgery, where the technique can influence the timing, rather than solely the magnitude, of oxidative changes. PNP and CO_2_ insufflation can alter tissue perfusion and trigger ischemia–reperfusion-type mechanisms, which are more likely to manifest later rather than immediately after the end of the procedure [21,22,51]. Notably, in both groups, TAS decreased markedly at T3, accompanied by pronounced changes in OSI. In the LAPOVE group, the OSI initially decreased by 43% at T2 and subsequently increased by 58% at T3, reflecting the dynamic nature of postoperative oxidative stress.

The results of this study suggest that laparoscopic OVE is less traumatic and is associated with lower intraoperative OS compared with open ovariectomy. However, the reduction in TAS and the increase in OSI 2 h after surgery in the LAPOVE group indicate that laparoscopic OVE may induce postoperative tissue stress similar to that observed after open OVE. Damolin et al. reported similar oxidative stress between video-assisted and open OHE. Although differences in surgical technique were considered, the authors identified CO_2_ PNP as a potential factor contributing to increased OS in video-assisted procedures. This mechanism may explain the comparable OS observed between the two techniques, despite the typically lower degree of tissue trauma associated with laparoscopy [32]. In the current study, potential factors contributing to postoperative changes in OS parameters in the LAPOVE group may also include CO_2_ PNP, which was established and maintained within an IAP range of 10–12 mmHg throughout the surgical procedure. Low postoperative cortisol concentrations may indicate reduced tissue stress associated with the laparoscopic approach. Therefore, surgical stress might not have been a major contributing factor to the observed changes in oxidative stress parameters in the LAPOVE group.

Studies investigating the effects of cortisol on OS in dogs undergoing surgical procedures, representing acute stress, are currently lacking. In the present study, serum cortisol levels showed a strong negative correlation with the TOS and OSI in the LPTOVE group. These findings suggest that an adequate cortisol response during and after open OVE may play a protective role against surgery-induced OS. Higher cortisol levels appeared to be protective against OS, whereas lower cortisol levels were associated with a greater oxidant burden [44]. This may support the concept that cortisol acts as an adaptive, redox-stabilizing hormone during acute stress. However, further targeted research is required to elucidate the underlying mechanisms.

Several studies have reported no or only minimal significant differences in heart rate, respiratory rate, and end-tidal CO_2_ (EtCO_2_) between laparoscopic and open ovariectomy [30,33,52,53]. In the present study, HR increased during anesthesia in both the LPTOVE and LAPOVE groups, remaining within reference ranges throughout. RR increased in the LAPOVE group but remained stable in the LPTOVE group, also staying within reference ranges. The findings of the present study are consistent with those reported by Costa et al., who observed no increase greater than 20% in heart rate and respiratory rate during surgery [30]. Furthermore, in this study, sedation in dogs from both groups was achieved using a combination of methadone, a full μ-opioid receptor agonist, and dexmedetomidine, an α_2_-adrenergic receptor agonist. Multiple studies have shown that the combination of methadone and dexmedetomidine significantly reduces the heart rate and moderately affects the respiratory rate in dogs during surgical procedures [54,55,56]. Nishimura et al. found that the combination of dexmedetomidine and methadone significantly reduced the heart and respiratory rates from 15 to 120 min, whereas Bustamante et al. observed decreased heart and respiratory rates for 45 min, with no significant differences in respiratory rate between treatment groups [55,56]. In this study, the time required for the removal of the first and second ovaries was not recorded. During these procedures, manipulation of the suspensory ligament could have resulted in increases in heart and respiratory rates, potentially influencing the measured parameters.

In contrast to previous studies, we found significant differences in RR at t = 15 min and t = 20 min between groups, with higher values observed in the LPTOVE group compared with the LAPOVE group. In addition, in the LPTOVE group, the median cortisol level was 9% above the upper limit of the reference range. Nonetheless, the available studies do not support a direct association between cortisol levels above the reference range and increased RR in healthy adult dogs [57,58,59]. In order to obtain the most accurate cortisol and OS measurements, efforts were made to minimize factors that could influence stress responses. Therefore, vital parameters were not assessed immediately prior to sedation or 15 min after the administration of sedative agents. It is possible that the respiratory rate of animals in the LPTOVE group was higher at the beginning of the study and may have influenced the obtained results. However, during the clinical examination conducted for animal selection, the respiratory rates of the dogs in both groups were within the physiological reference ranges. Other significant differences in RR between groups were observed at t = 45 min and t = 50 min, with higher values recorded in the LPTOVE group compared with the LAPOVE group. As mentioned previously, in this study, the time required for the removal of the first and second ovaries was not recorded. However, elevations in HR and RR in the LPTOVE group were observed at similar time points, which may suggest a stress response associated with tissue trauma. Furthermore, the cortisol levels in the LPTOVE group were 51% higher at the end of surgery compared with the beginning, whereas the cortisol levels in the LAPOVE group remained relatively stable. These findings suggest that, at these time points, laparoscopic OVE may have been less traumatic and induced a lower stress response compared with open OVE.

Reportedly, EtCO_2_ values remained relatively stable throughout laparoscopic OVE in dogs, exhibiting only mild stage-dependent variations [33,52,53]. During laparoscopic surgery, CO_2_ is insufflated into the abdominal cavity to establish PNP. Absorption of CO_2_ through the peritoneum may result in hypercapnia, potentially leading to clinically relevant acid–base disturbances [21]. In the present study, significantly higher EtCO_2_ values were observed at the last three anesthesia time points (t = 1 h 00 min, t = 1 h 05 min, and t = 1 h 10 min) in the LAPOVE group compared with the LPTOVE group. Fernandez-Martin et al. reported that mechanical ventilation may effectively compensate for hypercapnia resulting from CO_2_ absorption during PNP [53]. In contrast, Merlin et al. observed significant temporal changes in respiratory parameters, including a trend toward increased end-tidal CO_2_ after the insufflation of CO_2_, although 20 of 21 dogs maintained acceptable values without the need for assisted ventilation [52].

Hypercapnia and acid–base disturbances were positively associated with higher intra-abdominal pressure during PNP [19,20]. Multiple studies support the use of an IAP of 8–12 mmHg in dogs undergoing laparoscopic surgery [22,60,61,62]. Fernandez-Martin et al. and Naghavi et al. used IAP of 8–10 mmHg to maintain PNP, whereas Costa et al. used 12 mmHg. However, no differences in EtCO_2_ were observed in any of the studies [30,33,53]. Our findings were partially consistent with those reported by Merlin et al., in which an IAP of 10 mmHg was used for PNP maintenance. However, mechanical ventilation was not used in their study, in contrast to our methodology [52].

Variations in anesthesia depth may affect end-tidal CO_2_ measurements [63]. Previous research on the MAC requirements of sevoflurane (vaporizer setting 2%) reported significantly higher F_E_’Sevo concentrations during open ovariectomy compared with laparoscopic surgery [53]. Conversely, Costa et al. maintained anesthesia using 3–4% sevoflurane [30]. Both studies, in contrast to our results, observed no changes in EtCO_2_. The findings of this study suggest that ventilation may not have been sufficient, that IAP may have been too high, or that the sevoflurane concentration may have been excessive for dogs undergoing laparoscopic OVE. However, further studies are needed to clarify these observations.

This study has several limitations. The sample size was relatively small (10 dogs per group), which may have limited the statistical power of the analyses, particularly for oxidative stress parameters. Pain scores were not evaluated, although their assessment alongside cortisol measurements could have provided additional information on differences in perioperative pain between the surgical techniques. Furthermore, potential interactions and multifactorial environmental factors affecting oxidant and antioxidant values were not considered and may have influenced the oxidative stress results. The present study also did not investigate whether elevated EtCO_2_ values may have affected oxidative stress through mechanisms such as mild respiratory acidosis or local ischemia–reperfusion associated with pneumoperitoneum. Lastly, the animals’ reproductive history was not recorded, and potential effects on hormonal balance and oxidative stress responses may therefore have influenced the evaluated biomarkers.

Future studies with larger sample sizes are needed to confirm the findings of the present study and to improve the statistical power of the analyses. The inclusion of validated pain assessment scales alongside cortisol measurements may provide a more comprehensive evaluation of perioperative stress responses associated with different surgical techniques. Additionally, future research should consider evaluating a broader panel of oxidative stress biomarkers and longer postoperative monitoring periods to better characterize oxidative stress dynamics following laparoscopic and open ovariectomy. The potential influence of reproductive history, environmental factors, and pneumoperitoneum parameters should also be investigated to further clarify their role in oxidative stress responses during laparoscopic procedures.

## 5. Conclusions

Lower postoperative cortisol concentrations and reduced intraoperative oxidative stress indicate that laparoscopic ovariectomy induces a lower response to surgical stress compared with open ovariectomy. The observed increase in postoperative oxidative stress and EtCO_2_ at the end of laparoscopic ovariectomy may be related to pneumoperitoneum. Overall, these findings support laparoscopic ovariectomy as a less traumatic alternative to open surgery in dogs. The careful management of pneumoperitoneum and anesthetic parameters is recommended to minimize postoperative stress after laparoscopic procedures.

## Figures and Tables

**Table 1:** Correlation between cortisol and oxidative stress parameters in dogs undergoing laparotomy (LPTOVE) and laparoscopic ovariectomy (LAPOVE) using the same anesthetic protocol.

	Cortisol, µg/dL	TOS, μmol H_2_O_2_ Eq/L				TAS, mmol Trolox Eq/L				OSI, a.u.			
		T0	T1	T2	T3	T0	T1	T2	T3	T0	T1	T2	T3
LPTOVE	T0	−0.201	0.079	−0.600	0.139	0.224	−0.042	0.018	−0.418	−0.358	−0.018	−0.273	0.479
	T1	−0.468	−0.236	−0.673 ^*a*^	−0.018	0.200	−0.333	−0.115	−0.345	−0.442	0.261	−0.333	0.224
	T2	−0.578	−0.588	−0.248	−0.576	0.152	0.309	0.394	0.261	−0.261	−0.358	−0.721 ^*a*^	−0.564
	T3	−0.778	−0.685	−0.588	−0.321	0.309	−0.127	−0.006	−0.018	−0.527	0.042	−0.418	−0.212
LAPOVE	T0	0.055	−0.103	−0.248	0.442	−0.527	−0.152	−0.382	−0.030	0.115	−0.127	0.067	0.079
	T1	−0.042	0.127	−0.115	0.576	−0.806	−0.261	−0.261	−0.188	0.236	0.042	0.200	0.285
	T2	−0.612	−0.103	−0.079	0.018	0.006	−0.127	0.139	0.042	−0.394	0.139	−0.115	−0.370
	T3	−0.479	0.261	0.285	0.382	0.042	0.139	0.248	0.042	−0.236	−0.067	0.273	0.006

Values are expressed as correlation coefficients (r). (T0)—blood sample taken prior to sedation; (T1)—blood sample taken prior to surgical incision; (T2)—prior to surgical closure in the LPTOVE group, and prior to the deflation of CO_2_ in the LAPOVE group; (T3)—blood sample taken 2 h after the surgery. ^*a*^ indicates significant correlation between cortisol and oxidative stress parameters within the LPTOVE group (*p* < 0.05).

**Table 2:** Heart rate (HR) at different time points in dogs undergoing laparotomy (LPTOVE) and laparoscopic ovariectomy (LAPOVE) using the same anesthetic protocol.

Time (h:min)	LPTOVE		LAPOVE		Confidence Between Groups (*p*)
	Median	IQR: 25th–75th Percentiles	Median	IQR: 25th–75th Percentiles	
00:15	60	54–65	62	60–76	0.322
00:20	65	54–70	66	60–78	0.324
00:25	65	56–76	65	60–78	0.470
00:30	62	58–76	65	60–80	0.253
00:35	60	54–68	70 ^*a*’^ **^(*p* = 0.028)^**	64–80	0.053
00:40	64	60–72	71 ^*a*’^ **^(*p* = 0.016)^**	66–86	0.225
00:45	70 ^*a*^ **^(*p* = 0.013)^**	60–72	72 ^*a*’^ **^(*p* = 0.014)^**	68–86	0.254
00:50	69 ^*a*^ **^(*p* = 0.015)^**	60–72	72 ^*a*’^ **^(*p* = 0.008)^**	70–86	0.148
00:55	66 ^*a*^ **^(*p* = 0.012)^**	60–78	73 ^*a*’^ **^(*p* = 0.013)^**	62–86	0.288
01:00	67 ^*a*^ **^(*p* = 0.037)^**	56–76	74 ^*a*’^ **^(*p* = 0.008)^**	70–88	0.197
01:05	64 ^*a*^ **^(*p* = 0.049)^**	58–80	76 ^*a*’^ **^(*p* = 0.008)^**	70–87	0.095
01:10	63 ^*a*^ **^(*p* = 0.049)^**	58–80	79 ^*a*’^ **^(*p* = 0.007)^**	72–90	0.058

^*a*^ indicates significant differences from t = 15 min within the LPTOVE group and ^*a*’^ within the LAPOVE group (*p* < 0.05). Significant *p* values are shown in bold.

**Table 3:** Respiratory rate (RR) at different time points in dogs undergoing laparotomy (LPTOVE) and laparoscopic ovariectomy (LAPOVE) using the same anesthetic protocol.

Time (h:min)	LPTOVE		LAPOVE		Confidence Between Groups (*p*)
	Median	IQR: 25th–75th Percentiles	Median	IQR: 25th–75th Percentiles	
00:15	13 ^c^	10–20	10 ^c^	10–10	**0.009**
00:20	12 ^c^	10–20	10 ^c^	8–10	**0.008**
00:25	11	10–20	10	10–10	0.052
00:30	12	10–20	10	8–12	0.103
00:35	11	10–18	10	8–12	0.214
00:40	12	10–18	11	8–12	0.175
00:45	12 ^c^	10–18	10 ^c^	10–12	**0.049**
00:50	13 ^c^	12–23	10 ^*a*’^ **^(*p* = 0.034)^**^,^^c^	10–12	**0.036**
00:55	12	10–20	12 ^*a*’^ **^(*p* = 0.039)^**	10–12	0.304
01:00	12	10–22	12 ^*a*’^ **^(*p* = 0.039)^**	10–12	0.304
01:05	13	10–20	12 ^*a*’^ **^(*p* = 0.020)^**	10–12	0.126
01:10	13	10–20	11 ^*a*’^ **^(*p* = 0.046)^**	10–12	0.122

^*a*’^ indicates significant differences from t = 15 min within the LAPOVE group (*p* < 0.05). ^c^ indicates significant differences between the LPTOVE and LAPOVE groups at the same time points (*p* < 0.05). Significant *p* values are shown in bold.

**Table 4:** End-tidal carbon dioxide (EtCO_2_) at different time points in dogs undergoing laparotomy (LPTOVE) and laparoscopic ovariectomy (LAPOVE) using the same anesthetic protocol.

Time (h:min)	LPTOVE		LAPOVE		Confidence Between Groups (*p*)
	Median	IQR: 25th–75th Percentiles	Median	IQR: 25th–75th Percentiles	
00:15	-	-	-	-	-
00:20	44	39–53	43	42–45	0.733
00:25	44	36–52	43	41–44	0.704
00:30	45.5	39–52	42.5	41–44	0.495
00:35	46.5	39–54	44	42–45	0.879
00:40	44.5	42–51	44	43–47	0.939
00:45	45	42–50	45.5	43–47	0.790
00:50	44	42–51	45.5	43–47	0.704
00:55	44.5	42–50	49	44–49	0.197
01:00	42.5 ^c^	39–48	50.5 ^c^	49–52	**0.002**
01:05	43 ^c^	39–47	51.5 ^c^	49–52	**<0.001**
01:10	43 ^c^	39–46	51 ^c^	50–52	**<0.001**

^c^ indicates significant differences between the LPTOVE and LAPOVE groups at the same time points (*p* < 0.05). Significant *p* values are shown in bold.

## Data Availability

The data presented in this study are available on request from the corresponding author due to privacy restrictions.

## Abbreviations

The following abbreviations are used in this manuscript:

OVE	Ovariectomy
OHE	Ovariohysterectomy
PNP	Pneumoperitoneum
IAP	Intra-abdominal pressure
LPTOVE	Laparotomy ovariectomy
LAPOVE	Laparoscopic ovariectomy
OS	Oxidative stress
TOS	Total oxidant status
TAS	Total antioxidant status
OD	Optical density
OSI	Oxidative stress index
HR	Heart rate
RR	Respiratory rate
EtCO_2_	End-tidal CO_2_
CO_2_	Carbon dioxide

## References

[1] Schwarzkopf I., Van Goethem B., Vandekerckhove P.M., de Rooster H. (2015). Vessel sealing versus suture ligation for canine ovarian pedicle haemostasis: A randomised clinical trial. Vet. Rec..

[2] Watts J. (2018). The use of bipolar electrosurgical forceps for haemostasis in open surgical ovariectomy of bitches and queens and castration of dogs. J. Small Anim. Pract..

[3] Urfer S.R., Kaeberlein M. (2019). Desexing dogs: A review of the current literature. Animals.

[4] Dupré G., Fiorbianco V., Skalicky M., Gültiken N., Ay S.S., Findik M. (2009). Laparoscopic ovariectomy in dogs: Comparison between single portal and two-portal access. Vet. Surg..

[5] Nylund A.M., Drury A., Weir H., Monnet E. (2017). Rates of intraoperative complications and conversion to laparotomy during laparoscopic ovariectomy performed by veterinary students: 161 cases (2010–2014). J. Am. Vet. Med. Assoc..

[6] Bakhtiari J., Khalaj A.R., Aminlou E., Niasari-Naslaji A. (2012). Comparative evaluation of conventional and transvaginal laparoscopic ovariohysterectomy in dogs. Vet. Surg..

[7] Corriveau K.M., Giuffrida M.A., Mayhew P.D., Runge J.J. (2017). Outcome of laparoscopic ovariectomy and laparoscopic-assisted ovariohysterectomy in dogs: 278 cases (2003–2013). J. Am. Vet. Med. Assoc..

[8] Cicirelli V., Lacalandra G.M., Aiudi G.G. (2022). The effect of splash block on the need for analgesia in dogs subjected to video-assisted ovariectomy. Vet. Med. Sci..

[9] Cassata G., Palumbo V.D., Cicero L., Damiano G., Maenza A., Migliazzo A., Di Paola G., Vicari D., Fazzotta S., Lo Monte A.I. (2016). Laparotomic vs laparoscopic ovariectomy: Comparing the two methods. The ovariectomy in the bitch in laparoscopic era. Acta Biomed..

[10] Devitt C.M., Cox R.E., Hailey J.J. (2005). Duration, complications, stress, and pain of open ovariohysterectomy versus a simple method of laparoscopic-assisted ovariohysterectomy in dogs. J. Am. Vet. Med. Assoc..

[11] Davidson E.B., Moll H.D., Payton M.E. (2004). Comparison of laparoscopic ovariohysterectomy and ovariohysterectomy in dogs. Vet. Surg..

[12] Culp W.T.N., Mayhew P.D., Brown D.C. (2009). Effect of laparoscopic versus open ovariectomy on postsurgical activity in small dogs. Vet. Surg..

[13] Charlesworth T.M., Sanchez F.T. (2019). A comparison of the rates of postoperative complications between dogs undergoing laparoscopic and open ovariectomy. J. Small Anim. Pract..

[14] Hancock R.B., Lanz O.I., Waldron D.R., Duncan R.B., Broadstone R.V., Hendrix P.K. (2005). Comparison of postoperative pain after ovariohysterectomy by harmonic scalpel-assisted laparoscopy compared with median celiotomy and ligation in dogs. Vet. Surg..

[15] Shariati E., Bakhtiari J., Khalaj A., Niasari-Naslaji A. (2014). Comparison between two portal laparoscopy and open surgery for ovariectomy in dogs. Vet. Res. Forum.

[16] Mayhew P.D., Freeman L., Kwan T., Brown D.C. (2012). Comparison of surgical site infection rates in clean and clean-contaminated wounds in dogs and cats after minimally invasive versus open surgery: 179 cases (2007–2008). J. Am. Vet. Med. Assoc..

[17] Di Bella C., Lacitignola L., Grasso S., Centonze P., Greco A., Ostuni R., Crovace A., Staffieri F. (2018). An alveolar recruitment maneuver followed by positive end-expiratory pressure improves lung function in healthy dogs undergoing laparoscopy. Vet. Anaesth. Analg..

[18] Kim M.C., Lee J.Y. (2014). Comparison of oxidative stress status in dogs undergoing laparoscopic and open ovariectomy. J. Vet. Med. Sci..

[19] Scott J., Singh A., Valverde A. (2020). Pneumoperitoneum in veterinary laparoscopy: A review. Vet. Sci..

[20] Umano G.R., Delehaye G., Noviello C., Papparella A. (2021). The “dark side” of pneumoperitoneum and laparoscopy. Minim. Invasive Surg..

[21] Fukushima F.B., Malm C., Andrade M.E.J., Oliveira H.P., Melo E.G., Caldeira F.M.C., Gheller V.A., Palhares M.S., Macedo S.P., Figueiredo M.S. (2011). Cardiorespiratory and blood gas alterations during laparoscopic surgery for intrauterine artificial insemination in dogs. Can. Vet. J..

[22] Lee J.Y., Choi S.H. (2015). Evaluation of total oxidant and antioxidant status in dogs under different CO_2_ pneumoperitoneum conditions. Acta Vet. Scand..

[23] Lee Y., Yeum K., Song B.C. (2015). Impact of volatile anesthetics on oxidative stress and inflammation. Biomed. Res. Int..

[24] Tomsič K., Nemec Svete A. (2022). A mini-review of the effects of inhalational and intravenous anesthetics on oxidative stress in dogs. Front. Vet. Sci..

[25] Mittal M., Siddiqui M.R., Tran K., Reddy S.P., Malik A.B. (2014). Reactive oxygen species in inflammation and tissue injury. Antioxid. Redox Signal..

[26] Dissemond J., Goos M., Wagner S.N. (2002). The role of oxidative stress in the pathogenesis and therapy of chronic wounds. Hautarzt.

[27] Azizi S., Kazemi Mehrjerdi H., Zaeemi M. (2025). The protective role of melatonin and agomelatine against oxidative stress following laparoscopic ovariectomy in dogs. BMC Vet. Res..

[28] Soneja A., Drews M., Malinski T. (2005). Role of nitric oxide, nitroxidative and oxidative stress in wound healing. Pharmacol. Rep..

[29] Milech V., de Oliveira J.S., de Ataide M.A.W., Coradini G.P., Mann T.R., Sarturi V.Z., Hartmann H.., Linhares M.T., Antunes B.M., De Andrade C.M. (2021). Effects of heated pneumoperitoneum on inflammation, oxidative stress, and peritoneal histology in female dogs that underwent video-assisted ovariohysterectomy. Vet. Med. Int..

[30] Costa G.L., Leonardi F., Licata P., Porcino M., de Paoli F., Iannelli D., Bruno F., Macrì F., Iannelli N.M. (2025). Ovariectomy in canine surgical medicine: A comparative analysis of surgical approaches and the nociceptive, inflammatory, and oxidative stress responses. Animals.

[31] Kumari A., Tiwary R., Guha S.K., Kumar R., Kumar R. (2022). Oxidative stress and antioxidant activity in female dogs undergoing laparoscopic and open elective ovariectomy. Indian J. Anim. Sci..

[32] Dalmolin F., Rubio C.P., Furlanetto C.S., Steffens R., Hadi N.I.I.A., da Silva A.D.L., Tomazi P., Antunes B.M., Elias F., Schmidt E.M.S. (2024). Changes in biomarkers of inflammation and oxidative status in dogs subjected to celiotomy or video-assisted ovariohysterectomy. Vet. Sci..

[33] Naghavi R., Kazemi Mehrjerdi H., Heidarpour M. (2025). Assessment of pain, vital parameters and oxidative stress markers in dogs after celiotomy and three-port laparoscopic ovariectomy. Vet. Med. Sci..

[34] Lee J.Y., Won H.S., Hwang H.K., Jeong S.M., Kim M.C. (2013). Evaluation of the systemic oxidative stress status during major orthopedic surgery in dogs: A clinical study. J. Vet. Clin..

[35] Alipour F., Emami M.R., Mohri M. (2018). Endocrine and oxidative stress characteristics in different anesthetic methods during pneumoperitoneum in dogs. Comp. Clin. Pathol..

[36] Prete A., Yan Q., Al-Tarrah K., Akturk H.K., Prokop L.J., Alahdab F., Foster M.A., Lord J.M., Karavitaki N., Wass J.A. (2018). The cortisol stress response induced by surgery: A systematic review and meta-analysis. Clin. Endocrinol..

[37] Kumar R., Bishnoi P., Tanwar M., Jhirwal S.K., Kumari A. (2023). Haemato-biochemical changes after laparoscopic and conventional ovariohysterectomy in dogs. Indian J. Vet. Sci. Biotechnol..

[38] Okur D.T., Polat B. (2021). Comparison of the postoperative outcome of the three-port laparoscopic ovariectomy and conventional open ovariectomy methods in dogs. Thai J. Vet. Med..

[39] Sherin S.S., Saikia B., Konwar B., Ahmed F.A., Chaudhary J.K., Singh D.Y., Lalhmangaihzuala M. (2019). Comparison of ovariectomy by laparoscopic and conventional open method in dogs. Int. J. Livest. Res..

[40] Karić L., Janjić F., Spariosu K., Davitkov D., Krstić V., Kovačević Filipović M., Radaković M. (2024). (Un)tying the knot: Oxidative stress, inflammatory markers, and lipid status in dogs with hypercortisolism. Animals.

[41] Chen H., Liu Y., Deng X., Lin Y., Liu H., Zhu S., Kastelic J.P., Zhou X., Han B. (2025). Canine Cushing syndrome elevates oxidative stress that is attenuated by trilostane. Am. J. Vet. Res..

[42] Kim H.C., Yonezawa T., Maeda S., Tamahara S., Matsuki N. (2022). Increases in serum carbonylated protein levels of dogs with hypercortisolism. Endocr. J..

[43] Soares F.A.C., Filho N.A.K., Beretta B.F.S., Linden T.S., Pöppl A.G., González F.H.D. (2021). Thiobarbituric acid reactive substances in dogs with spontaneous hypercortisolism. Domest. Anim. Endocrinol..

[44] Birnie-Gauvin K., Peiman K.S., Larsen M.H., Aarestrup K., Willmore W.G., Cooke S.J. (2017). Short-term and long-term effects of transient exogenous cortisol manipulation on oxidative stress in juvenile brown trout. J. Exp. Biol..

[45] Lee J.Y. (2012). Oxidative stress due to anesthesia and surgical trauma and comparison of the effects of propofol and thiopental in dogs. J. Vet. Med. Sci..

[46] Baysal E., Taysi S., Aksoy N., Uyar M., Celenk F., Karatas Z.A., Tarakcioglu M., Bilinç H., Mumbuç S., Kanlikama M. (2012). Serum paraoxonase, arylesterase activity and oxidative status in patients with obstructive sleep apnea syndrome (OSAS). Eur. Rev. Med. Pharmacol. Sci..

[47] Burbaitė E., Čechovičienė S., Sarapinienė I., Karvelienė B., Riškevičienė V., Daunoras G., Juodžentė D. (2024). Effects of medetomidine–butorphanol and medetomidine–buprenorphine on oxidative stress and vital parameters in dogs undergoing ovariohysterectomy. Animals.

[48] Hasson H.M. (2001). Open laparoscopy as a method of access in laparoscopic surgery. Gynecol. Endosc..

[49] Szczubial M., Kankofer M., Bochniarz M., Dąbrowski R. (2015). Effects of ovariohysterectomy on oxidative stress markers in female dogs. Reprod. Domest. Anim..

[50] Thomas A., Karayannopoulou M., Anagnostou T., Psalla D., Ioannou K., Ginoudis A., Savvas I., Pardali D. (2025). Study of oxidant/antioxidant profile in dogs with mammary cancer undergoing mastectomy, during the peri-operative period. Vet. Sci..

[51] Nickkholgh A., Barro-Bejarano M., Liang R., Zorn M., Mehrabi A., Gebhard M.M., Büchler M.W., Gut C.N., Schemmer P. (2008). Signs of reperfusion injury following CO_2_ pneumoperitoneum: An in vivo microscopy study. Surg. Endosc..

[52] Merlin T., Cinti F., Charlesworth T.M. (2022). Healthy nonobese bitches maintain acceptable spontaneous ventilation during laparoscopic ovariectomies. J. Am. Vet. Med. Assoc..

[53] Valiño-Cultelli V., Fernández-Martín S., González-Cantalapiedra A. (2022). Laparoscopic versus open ovariectomy in bitches: Changes in cardiorespiratory values, blood parameters, and sevoflurane requirements associated with the surgical technique. Animals.

[54] Cardoso C.G., Marques D.R., da Silva T.H., de Mattos-Junior E. (2014). Cardiorespiratory, sedative and antinociceptive effects of dexmedetomidine alone or in combination with methadone, morphine or tramadol in dogs. Vet. Anaesth. Analg..

[55] Bustamante R., Canfrán S., Gómez de Segura I.A. (2024). Clinical evaluation of the sedative, antinociceptive and cardiorespiratory effects of intranasal dexmedetomidine combined with methadone in healthy dogs. Vet. J..

[56] Nishimura L.T., Auckburally A., Santilli J., Vieira B.H.B., Garcia D.O., Honsho C.S., de Mattos-Junior E. (2018). Effects of dexmedetomidine combined with commonly administered opioids on clinical variables in dogs. Am. J. Vet. Res..

[57] Naddaf H., Varzi H., Falah S. (2014). Effects of xylazine-ketamine anesthesia on plasma levels of cortisol and vital signs during laparotomy in dogs. Open Vet. J..

[58] Riggio G., Borrelli C., Campera M., Gazzano A., Mariti C. (2022). Physiological indicators of acute and chronic stress in securely and insecurely attached dogs undergoing a strange situation procedure (SSP): Preliminary results. Vet. Sci..

[59] Tannus F.C.I., Rahal S.C., Oba E., Tsunemi M., Silva B.M., Almeida K.C., Santos I.F.C. (2021). Serum cortisol and clinical response to a single session of whole-body vibration in healthy adult dogs. Front. Vet. Sci..

[60] Maiti S.K., Dutta A., Varshney J., Kumar N. (2013). Effect of different carbon dioxide pressure gradients in capnoperitoneum for laparoscopic examination in dogs. World J. Laparosc. Surg..

[61] Ishizaki Y., Bandai Y., Shimomura K., Abe H., Ohtomo Y., Idezuki Y. (1993). Safe intraabdominal pressure of carbon dioxide pneumoperitoneum during laparoscopic surgery. Surgery.

[62] Parlier M., Thomson C.B., Rendahl A., Strelchik A., Baldo C., Eckman S.K., Krueger A., Gordon-Evans W.J. (2024). Prospective, randomized, clinical trial on the effects of laparoscopic insufflation pressures on portal pressures in dogs. Vet. Surg..

[63] Kruljic P., Nemec A., Vintar N., Butinar J. (2003). Relation between end-tidal and arterial carbon dioxide partial pressure during general anaesthesia with spontaneous breathing and controlled ventilation in dogs: An experimental study. Acta Vet..

